# Curcumin Nanoparticles Protect against Isoproterenol Induced Myocardial Infarction by Alleviating Myocardial Tissue Oxidative Stress, Electrocardiogram, and Biological Changes

**DOI:** 10.3390/molecules24152802

**Published:** 2019-08-01

**Authors:** Paul-Mihai Boarescu, Ioana Boarescu, Ioana Corina Bocșan, Raluca Maria Pop, Dan Gheban, Adriana Elena Bulboacă, Cristina Nicula, Ruxandra-Mioara Râjnoveanu, Sorana D. Bolboacă

**Affiliations:** 1 Department of Pathophysiology, Iuliu Haţieganu University of Medicine and Pharmacy Cluj-Napoca, Victor Babeş Street, no. 2-4, 400012 Cluj-Napoca, Romania; 2 Department of Medical Informatics and Biostatistics, Iuliu Haţieganu University of Medicine and Pharmacy Cluj-Napoca, Louis Pasteur Street, no. 6, 400349 Cluj-Napoca, Romania; 3 Department of Neurology, County Clinical Emergency Hospital of Cluj-Napoca, Victor Babes Street, no 43, 400012 Cluj-Napoca, Romania; 4 Department of Pharmacology, Toxicology and Clinical Pharmacology, Iuliu Haţieganu University of Medicine and Pharmacy Cluj-Napoca, Gheorghe Marinescu Street, no 23, 400337 Cluj-Napoca, Romania; 5 Department of Pathological Anatomy, Iuliu Haţieganu University of Medicine and Pharmacy Cluj-Napoca, Clinicilor Street, no 3-5, 400006 Cluj-Napoca, Romania; 6 Department of Ophthalmology, Iuliu Haţieganu University of Medicine and Pharmacy Cluj-Napoca, Clinicilor Street, no 3-5, 400006 Cluj-Napoca, Romania; 7 Department of Pneumology, Iuliu Haţieganu University of Medicine and Pharmacy Cluj-Napoca, B.P. Hasdeu Street, no. 6, 400371 Cluj-Napoca, Romania

**Keywords:** myocardial infarction, curcumin, nanoparticles, isoproterenol, oxidative stress

## Abstract

Curcumin from *Curcuma longa* is a nutraceutical compound reported to possess strong antioxidant activity that makes it a candidate for use in counteracting oxidative stress-induced damage. The effect of pre-treatment with curcumin nanoparticles (nC) compared to conventional curcumin (Cs) on blood pressure, electrocardiogram, and biological changes on isoproterenol (ISO)-induced myocardial infarction (MI) in rats had been investigated. The Cs doses of 150 and 200 mg/kg bw and all nC doses (100, 150 and 200 mg/kg bw) significantly reduced heart rate before ISO administration and prevented QRS complex enlargement after MI induction (*p* < 0.026). All doses of Cs and nC prevented prolongation of the QT and QT corrected (QTc) intervals, with better results for higher doses (*p* < 0.048). The nC solution had more significant results than Cs in all metabolic parameters assessed (lactate dehydrogenase, glycaemia, aspartate transaminase, and alanine transaminase, *p* < 0.009). nC was more efficient than Cs in limiting myocardial oxidative stress and enhancing antioxidative capacity (*p* < 0.004). Compared to Cs, nC better prevented myocardial damage extension, reduced interstitial oedema, and inflammation. Curcumin nanoparticles as compared to conventional curcumin exert better antioxidative effects. Moreover, nC better prevent cardiomyocytes damage, and electrocardiogram alterations, in the case of ISO-induced MI in rats.

## 1. Introduction

Myocardial infarction (MI) is a leading cause of morbidity and is the primary cause of death in developed countries, despite rapid advancements made in the treatment strategies of ischemic heart disease [1,2]. MI is an important acute disease of myocardial tissue caused by an imbalance between myocardial blood demand and the coronary blood delivery, leading to cardiac ischemia, degeneration of cardiomyocytes and eventually irreversible cardiac injury or death [3]. The first ultrastructural changes observed in the early 10–15 min after the onset of myocardial ischemia are decreased cellular glycogen, relaxed myofibrils, and sarcolemmal disruption [4]. In animal models of MI, biochemical evidence of myocardial cell death due to apoptosis can be identified within 10 min of induced myocardial ischemia [4]. Preclinical studies concluded that during ischemic myocardial damage, oxidative stress from reactive oxygen species plays an important role in the development, evolution and extension of MI [5,6]. Catecholamines at low concentrations exert a positive inotropic effect, so they are considered to have a beneficial effect in regulating the activity of the heart [7]. Even so, their administration in high doses or excessive releasing from the endogenous stores may lead to a depletion of the energy reserve of cardiomyocytes, resulting in severe biological and structural changes, and eventually to irreversible damage and death [7]. Isoproterenol (ISO) is a synthetic catecholamine and β-adrenergic receptor agonist, which after autooxidation, generates high levels of reactive free radicals [8]. ISO also alters tissue antioxidative defence systems including chemical scavengers and antioxidant molecules [8]. Administered in high doses it causes severe oxidative stress in the myocardium, resulting in infarction-like lesions [9]. It was demonstrated that oxidative stress produced by ISO is mediated through free radicals or reactive oxygen species, therefore, any agent known to possess strong antioxidant effects is believed to protect the myocardial cells from MI damage [9]. Using ISO for experimental induction of MI in animals is a well-established model to study the effects of several compounds with cardioprotective effects. Curcumin from *Curcuma longa* is a nutraceutical compound reported to possess strong antioxidant activity that makes it a candidate for use in counteracting oxidative stress-induced damage [10]. It has a long history for the treatment of several diseases such as cancers, digestive disorders, and infectious, liver, rheumatoid diseases, diabetes and atherosclerosis [11,12,13]. In spite of its beneficial effects, due to fast metabolism, rapid systemic elimination, low gastrointestinal absorption, low aqueous solubility, and alkaline pH degradation, curcumin has a decreased bioavailability, which may represent a limitation of its clinical usage [14]. Nanotechnology may represent a part of the future technology in drug production, since the use of nanoparticles for drug delivery purposes seems not only to enhance curcumin permeability and increased absorption, but also to offer a stronger resistance to metabolic processes [15,16,17,18,19]. Curcumin and curcumin nanoparticles demonstrated to reduce inflammation and serum oxidative marks in the case of ISO-induced MI [20].

Our study aimed to investigate the effects of pre-treatment with curcumin nanoparticles solution compared to conventional curcumin solution on blood pressure, electrocardiogram (ECG), and biological changes on ISO-induced MI in rats. The primary objectives were to find if curcumin nanoparticles are better than curcumin, and the best doses that reduce the effects of ISO. 

## 2. Results

No rat died during the follow-up, so the analysis was conducted on all seven rats in each experimental group. All *p* values are presented in tables provided in Supplementary Materials.

### 2.1. Blood Pressure

No differences between the groups, in systolic or diastolic blood pressures, were found at the beginning of the experiment (*p* > 0.10, Table 1). Neither curcumin solution (Cs) nor curcumin nano-particle solution (nC) influenced the blood pressure values before or after ISO administration (*p* > 0.10, Table 1).

### 2.2. Electrocardiogram Monitoring

**Figure 1:**
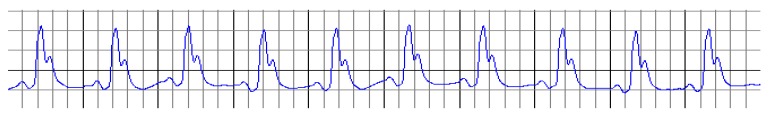
ECG record on day 0, at the beginning of the experiment.

Interpretation of the electrocardiograms (ECGs) of all groups, performed on day 0, is summarised in Table 2, and no differences were found between groups. A representative electrocardiogram (ECG) record for all groups is shown in Figure 1. P-values for comparisons between the study groups for electrocardiogram monitoring are presented in Supplementary Table S1. 

**Figure 2:**
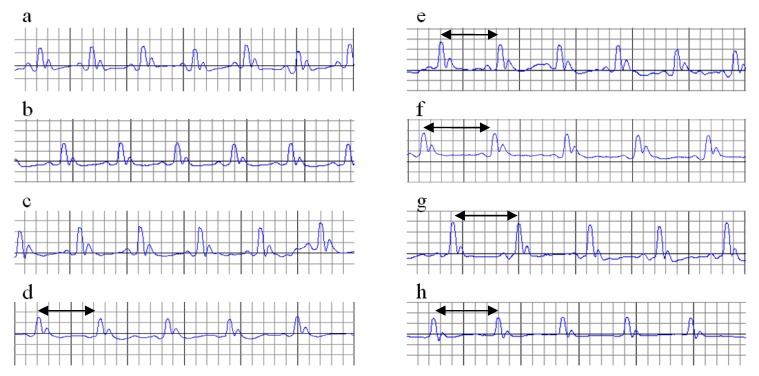
ECGs on day 12 (before ISO administration). Increased RR interval (black arrow). (**a**) C = Control; (**b**) MI-C = MI without any pre-treatment; (**c**) curcumin solution, as 100 mg/kg bw (Cs100), (**d**) curcumin solution, as 150 mg/kg bw (Cs150), (**e**) curcumin solution, as 200 mg/kg bw (Cs200); (**f**) curcumin nano-particle solution as 100 mg/kg bw (nC100), (**g**) curcumin nano-particle solution as 150 mg/kg bw (nC150), and (**h**) curcumin nano-particle solution as 200 mg/kg bw (nC200); ISO = isoproterenol.

Cs in the doses of 150 mg/kg bw and 200 mg/kg bw (*p* ≤ 0.025) and nC in all doses had an increased RR interval and reduced the heart rate before ISO administration (Table 3, Figure 2e–h), with significantly better results for nC compared to Cs (*p* = 0.0017). No other changes were identified in groups with Cs or nC before ISO administration. 

**Figure 3:**
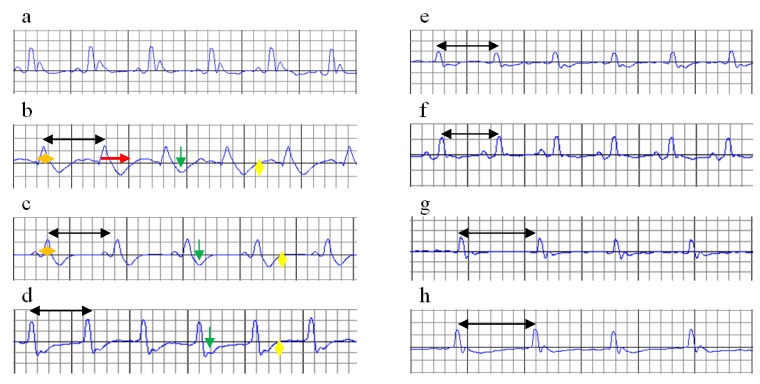
ECGs on day 15 (after ISO administration). Increased RR interval (black arrow), enlargement of the QRS complex (orange arrow), increased QT interval (red arrow), ST-segment depression (green arrow), T wave inversion (yellow arrow). (**a**) C = Control; (**b**) MI-C = myocardial infarction (MI) without any pre-treatment; (**c**) curcumin solution, as 100 mg/kg bw (Cs100), (**d**) curcumin solution, as 150 mg/kg bw (Cs150), (**e**) curcumin solution, as 200 mg/kg bw (Cs200); (**f**) curcumin nano-particle solution as 100 mg/kg bw (nC100), (**g**) curcumin nano-particle solution as 150 mg/kg bw (nC150), and (**h**) curcumin nano-particle solution as 200 mg/kg bw (nC200); ISO = isoproterenol.

ISO administration induced significant alterations in ECG recordings, such as decreased RR intervals, increased heart rate (HR), increased QT and QTc intervals and depression of the ST-segment associated with marked T wave inversion (Table 4, Figure 3b), which reflects an ISO-induced infarct-like lesion. ISO had no effect on the PR segment.

After ISO administration, Cs and nC in all doses increased the RR interval and reduced the heart rate, with better results for nC (*p* ≤ 0.0476), while efficacy is improved by increasing dose (*p* = 0.0017, Table 4, Figure 3c–h). Cs in highest doses and nC in all doses prevented enlargement of the QRS complex, with more significant results for nC (*p* ≤ 0.0022, Table 4, Figure 3e–h). No differences were found between the highest doses of Cs (*p* > 0.05) and the lowest doses of nC (*p* > 0.05). All doses of Cs and nC prevented prolongation of the QT and QTc intervals, with better results if the dose is increased (*p* ≤ 0.0476, Table 4, Figure 3c–h). nC, in all doses, better prevented prolongation of the QT and QTc intervals compared to Cs (*p* = 0.0017, Table 4, Figure 3c–h). Pre-treatment with Cs in the dose of 200 mg/kg bw and all doses of nC reduced ST depression and T waves inversion (*p* ≤ 0.0106, Table 4, Figure 3e–h). The greatest effect was obtained for nC in a dose of 200 mg/kg bw (*p* ≤ 0.0152, Table 4, Figure 3c–h).

### 2.3. Blood Samples and Serum Analysis 

**Figure 4:**
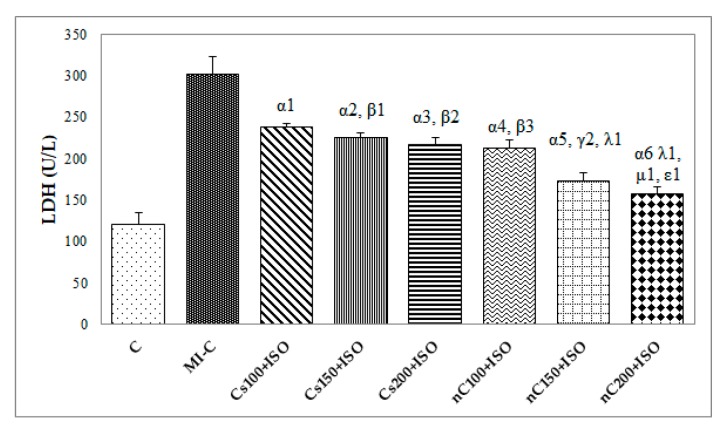
Serum levels of lactate dehydrogenase (LDH) by groups. C = control; MI-C = isoproterenol without any pre-treatment; Cs = curcumin solution, in doses of 100 mg/kg bw (Cs100), 150 mg/kg bw (Cs150), and 200 mg/kg bw (Cs200); nC = curcumin nanoparticle solution, in doses of 100 mg/kg bw (nC100), 150 mg/kg bw (nC150), and 200 mg/kg bw (nC200). The letter-number codes correspond to the *p*-values < 0.05: ^α^ as compared with MI-C, ^β^ as compared with CS100+ISO, ^γ^ as compared with CS150+ISO, ^λ^ as compared with nC100+ISO, ^μ^ as compared with nC150+ISO, ^ε^ as compared with Cs200+ISO.

**Figure 5:**
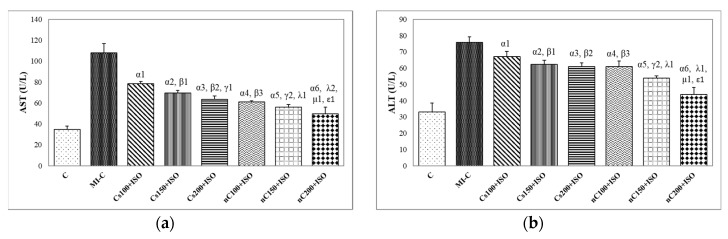
Serum levels of: (**a**) aspartate transaminase (AST) and (**b**) alanine transaminase (ALT) by groups. C = control; MI-C = isoproterenol without any pre-treatment; Cs = curcumin solution, in doses of 100 mg/kg bw (Cs100), 150 mg/kg bw (Cs150), and 200 mg/kg bw (Cs200); nC = curcumin nanoparticle solution, in doses of 100 mg/kg bw (nC100), 150 mg/kg bw (nC150), and 200 mg/kg bw (nC200). The letter-number codes correspond to the *p*-values < 0.05: ^α^ as compared with MI-C, ^β^ as compared with CS100+ISO, ^γ^ as compared with CS150+ISO, ^λ^ as compared with nC100+ISO, ^μ^ as compared with nC150+ISO, ^ε^ as compared with Cs200+ISO.

**Figure 6:**
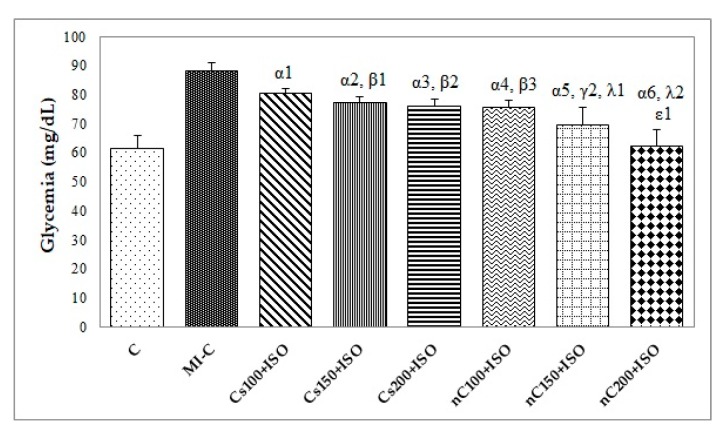
Serum levels of glycemia by groups. C = control; MI-C = isoproterenol without any pre-treatment; Cs = curcumin solution, in doses of 100 mg/kg bw (Cs100), 150 mg/kg bw (Cs150), and 200 mg/kg bw (Cs200); nC = curcumin nanoparticle solution, in doses of 100 mg/kg bw (nC100), 150 mg/kg bw (nC150), and 200 mg/kg bw (nC200). The letter-number codes correspond to the *p*-values < 0.05: ^α^ as compared with MI-C, ^β^ as compared with CS100+ISO, ^γ^ as compared with CS150+ISO, ^λ^ as compared with nC100+ISO, ^ε^ as compared with Cs200+ISO.

Our results showed increased serum levels of lactate dehydrogenase (LDH, Figure 4), aspartate transaminase (AST, Figure 5a), alanine transaminase (ALT, Figure 5b), and glycaemia (Figure 6) after the ISO administration. *p*-Values for comparisons between the study groups for serum analysis are presented in Supplementary Table S2. All doses of Cs and nC prevented the elevation of LDH, AST, ALT, and glycaemia with better results for nC compared to Cs (*p* ≤ 0.0127, Figure 4, Figure 5a,b, and Figure 6). The increase in dose of Cs and nC had better preventive effects on all blood serum parameters measured (*p* ≤ 0.0127, Figure 4, Figure 5a,b, and Figure 6). No statistical differences were found between the doses of 150 mg/kg bw and 200 mg/kg bw for glycaemia, neither for Cs, nor for nC (*p* > 0.05, Figure 6). The dose 150 mg/kg bw of Cs had the same effect as 200 mg/kg bw Cs on LDH, ALT and glycaemia (*p* > 0.05, Figure 4, Figure 5b and Figure 6).

### 2.4. Assessment of Oxidative Stress Parameters in Myocardial Tissue

**Figure 7:**
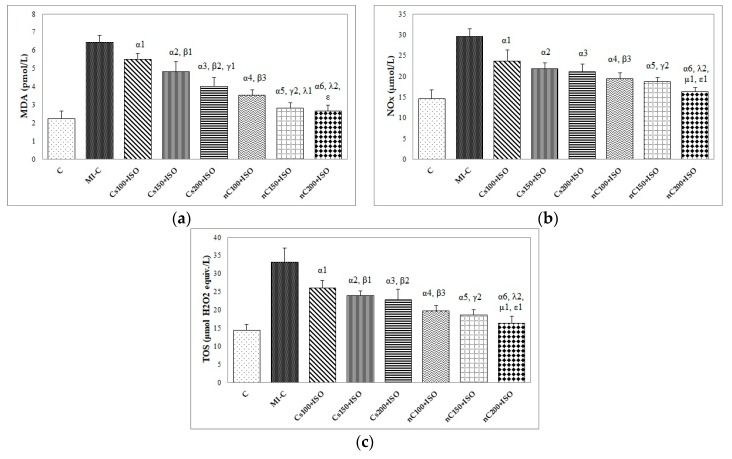
Distribution of oxidative stress intensity in the myocardial tissue: (**a**) nitric oxide (NOx), (**b**) malondialdehyde (MDA), and (**c**) total oxidative status (TOS) by groups. C = control; MI-C = isoproterenol without any pre-treatment; Cs = curcumin solution, in doses of 100 mg/kg bw (Cs100), 150 mg/kg bw (Cs150), and 200 mg/kg bw (Cs200); nC = curcumin nanoparticle solution, in doses of 100 mg/kg bw (nC100), 150 mg/kg bw (nC150), and 200 mg/kg bw (nC200); the letter-number codes correspond to the *p*-values < 0.05: ^α^ as compared with MI-C, ^β^ as compared with CS100+ISO, ^γ^ as compared with CS150+ISO, ^λ^ as compared with nC100+ISO, ^μ^ as compared with nC150+ISO, ^ε^ as compared with Cs200+ISO.

**Figure 8:**
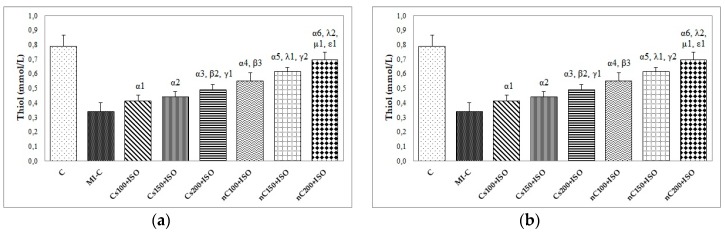
Distribution of antioxidant capacity in the myocardial tissue: (**a**) Thiol and (**b**) total antioxidant capacity (TAC) by groups. C = control; MI-C = isoproterenol without any pre-treatment; Cs = curcumin solution, in doses of 100 mg/kg bw (Cs100), 150 mg/kg bw (Cs150), and 200 mg/kg bw (Cs200); nC = curcumin nanoparticle solution, in doses of 100 mg/kg bw (nC100), 150 mg/kg bw (nC150), and 200 mg/kg bw (nC200). The letter-number codes correspond to the *p*-values < 0.05: ^α^ as compared with MI-C, ^β^ as compared with CS100+ISO, ^γ^ as compared with CS150+ISO, ^λ^ as compared with nC100+ISO, ^μ^ as compared with nC150+ISO, ^ε^ as compared with Cs200+ISO.

**Figure 9:**
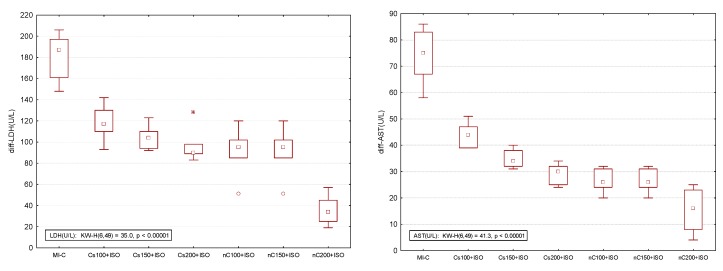

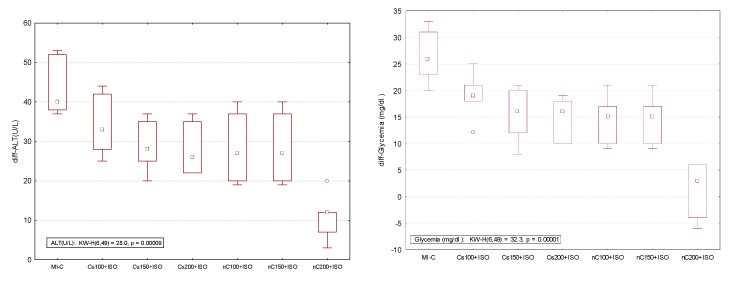
Differences relative to the control (no myocardial infarction, C group) of serum analysis represented by lactate dehydrogenase (LDH), aspartate transaminase (AST), alanine transaminase (ALT), and glycaemia. * extreme value; ^○^ outlier.

The inducement of MI resulted in an elevation of all oxidative stress markers in the myocardial tissue (Figure 7a–c). The increase in the dose of Cs had greater effects in preventing malondialdehyde (MDA) elevation (Figure 7a, *p* ≤ 0.0350) while nC in a dose of 150 mg/kg bw had the same effect as nC in a dose of 200 mg/kg bw (Figure 7a, *p* > 0.05). All doses of Cs had the same effect on nitric oxide (NOx), better results were obtained for nC compared to Cs (Figure 7b, *p* ≤ 0.0029). Highest doses of Cs had the same effect on the tissue level of total oxidative status (TOS) (Figure 7c, *p* > 0.05). For TOS, the same results were obtained for the lowest doses of nC (Figure 7c, *p* > 0.05), while the best results were obtained for the dose of 200 mg/kg bw (Figure 9c, *p* ≤ 0.047). After MI was induced, myocardial tissue levels in both thiol and total antioxidant capacity (TAC) values were significantly decreased (Figure 8a,b). Pre-treatment with any Cs dose prevented the reduction in thiol and TAC levels (Figure 8a,b). Both doses of 100 and 150 mg/kg bw Cs had a similar effect on Thiol levels (*p* > 0.05, Figure 8a). The increase in the dose of Cs and nC proved to be more efficient in preventing the decrease of myocardium level of TAC (Figure 8b, *p* ≤ 0.0250). For both thiol and TAC, better results were obtained for nC compared to Cs (Figure 8a,b, *p* ≤ 0.0017).

### 2.5. Overall Effect of Curcumin Nanoparticle Solution

**Figure 10:**
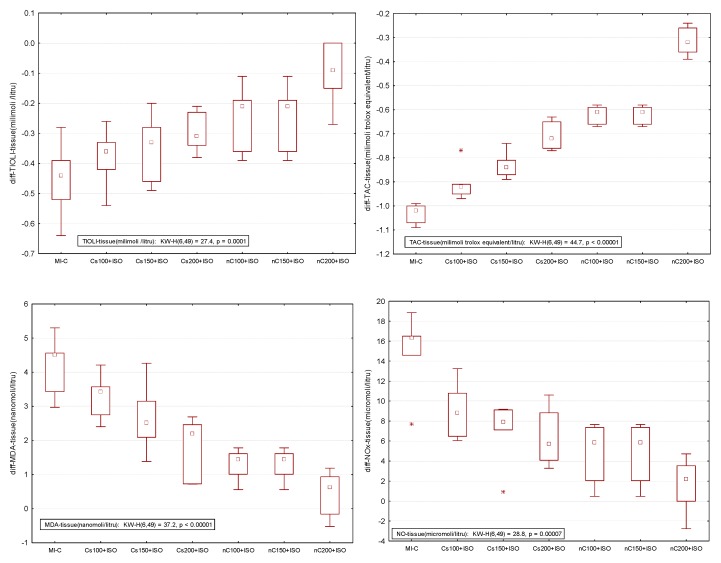

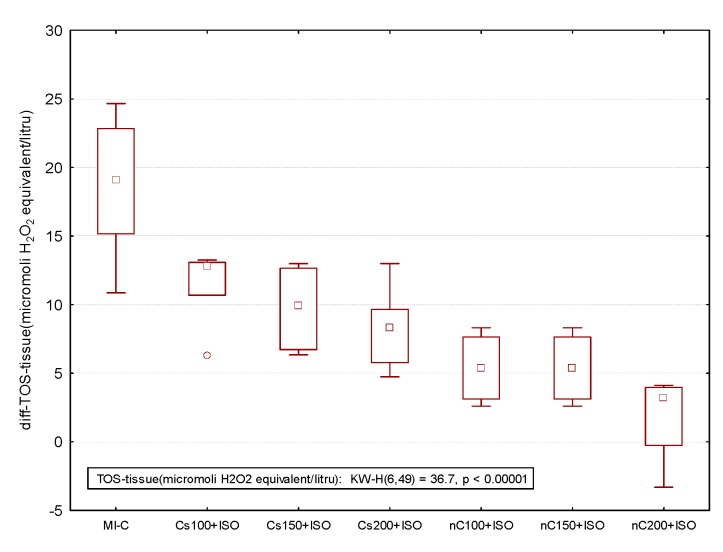
Differences relative to the control (no myocardial infarction, C group) of antioxidant capacity in the myocardial tissue: Thiol and total antioxidant capacity (TAC) by groups and of oxidative stress intensity in the myocardial tissue: nitric oxide (NOx), malondialdehyde (MDA), and total oxidative status (TOS) by groups. MI-C = isoproterenol without any pre-treatment; Cs = curcumin solution, in doses of 100 mg/kg bw (Cs100), 150 mg/kg bw (Cs150), and 200 mg/kg bw (Cs200); nC = curcumin nanoparticle solution, in doses of 100 mg/kg bw (nC100), 150 mg/kg bw (nC150), and 200 mg/kg bw (nC200). * extreme value; ^○^ outlier.

The overall effect of nC on the serum and oxidative stress on myocardial tissue investigated parameters is illustrated in Figure 9 and Figure 10. The nC200 was proved to be the most effective since the difference relative to the control group (no myocardial infarction, C group) was more close by no difference (zero). 

## 3. Discussion

On our experimental model, the highest dose of Cs and nC (200 mg/kg bw) proved to better prevent ECG and biological changes after ISO-induced MI. Curcumin nanoparticles had better improved effects than conventional curcumin in all of the studied parameters. 

Neither curcumin solution nor curcumin nanoparticles pre-treatment influenced systolic or diastolic blood pressures (Table 1). ISO was reported to reduce mean blood pressure in the case of acute and continuous administration and to increase it in case of chronic administration [21], but in our study at 24 h after administration of the last dose, it had no effect on blood pressure values. 

In our study pre-treatment with Cs 200 mg/kg bw, Cs 150 mg/kg bw and nC in all doses increased the RR interval and reduced HR (Table 3, Figure 2e–h). Kilinç et al. suggested that curcumin may be used in the treatment of pathologic tachycardia, as a specific bradycardic agent, since it decreases the heart rate without affecting the vitality of the heart [22]. The mechanism of how curcumin reduces heart rate is not known yet and needs further research. 

ISO administration led to a reduction of RR interval and increased HR (Table 4, Figure 3 b) since ISO is a β1 and β2 adrenoreceptor agonist and the activation of ß adrenergic receptors increases HR and force of contraction of the left ventricle [23]. ISO prolonged the QRS complex, increased QT and QTc intervals and induced depression of ST segment associated with marked T wave inversion, but had no effect on the PR segment (Table 4, Figure 3b). Prolongation of the QRS complex and QT, QTc intervals after ISO administration could indicate the slowing of ventricular conduction due to ISO cardiotoxic effect [24]. ST depression could be secondary to subendocardial ischemia and necrosis [24] since ISO is accelerating the heart rate inducing impairment in perfusion of the subendocardial myocardium of the left ventricle [25]. Generally, ST-interval depression and T-waves inversion are suggestive of acute myocardial infarction. On a cellular level, T-wave inversion was suggested to be secondary to changes in action potential duration in cardiomyocytes in a rat model after isoproterenol administration [26]. Pre-treatment with Cs in a dose of 200 mg/kg bw and all doses of nC had attenuated the ISO-induced ECG alterations. According to effects on the ECG pattern, nC offered a better cardioprotective effect then Cs. Nirmala et al. concluded that curcumin treatment to ISO administered rats had a stabilizing effect on the cellular membranes, explained by the fact that curcumin prevents the release of enzymes from nuclear, mitochondrial, microsomal and lysosomal fractions [27]. Pre-treatment with curcumin showed a protective effect against ECG alterations and prevented acute fatal complications of MI by protecting the structure of the cardiomyocytes membrane [27]. Some studies reported that curcumin blocks the human ether-a-go-go-related gene (hERG) coded potassium channels, which are responsible for the rapid phase of potassium channels repolarisation, leading to a prolongation of the QT interval [28,29]. A study performed by Ranjan et al. concluded that curcumin encapsulated in lipopolymer hybrid nanoparticle formulation protected against QT prolongation and overcame the limitations associated with curcumin, by increasing its bioavailability and stability thereby [30]. The study performed by Helson et al. showed that nanoparticles of curcumin with a liposomal formulation were able to prevent QTc prolongation [28]. ISO-induced ECG MI-like alterations such as reduction of the RR interval, increase of QT and QTc intervals, depression of the ST segment associated with marked T wave inversion, could be due to the secondary loss of action potential in the myocardial cell membrane, as a result of oxidative stress [23].

To our knowledge, this is the first study that evaluates the effects of curcumin nanoparticles pre-treatment compared to conventional curcumin on myocardial oxidative stress in ISO-induced MI in rats. The measured oxidative stress and antioxidant capacity parameters had a similar pattern in the blood [20] as in the in myocardial tissue after homogenization (Figure 7 and Figure 8), with higher expression in the myocardial tissue, as expected. Evaluating oxidative stress markers in the heart tissue, we found an elevation of myocardial levels of MDA, NOx, and TOS after MI induction, with lower levels in rats pre-treated with Cs and nC (Figure 7a–c). MDA is frequently used as a marker of reactive oxygen species (ROS) production since it is a stable lipid peroxidation end-product [31,32]. Increased levels of MDA in myocardial tissue of rats that received ISO indicate also increased lipid peroxidation. The reduction of MDA levels in myocardial tissue of rats pre-treated with Cs and nC (Figure 7a) suggests an inhibitory effect of the curcumin on lipid peroxidation. It was already proved that curcumin decreases the rate of tissue peroxidation while increasing antioxidant capacity after the occurrence of MI [33] and also that curcumin nanoparticles can better increase the antioxidant defence of the cardiomyocytes [34]. The tissular nitric oxide (NO) production, a biomarker of nitrooxidative stress, was evaluated by measuring the levels of the inorganic nitrites and nitrates (NOx), stable end metabolites of NO [35]. In MI, the increase of NO synthesis is secondary to the activation of the high-output inducible NOS/NO pathway [36]. This mechanism explains the high tissular levels of NOx in rats after ISO administration (Figure 7b). Rats with Cs had reduced levels of NOx compared to control rats with ISO (Figure 7b) since it was already reported that curcumin inhibits nitric oxide synthase activity [37]. Rats pretreated with nC had lower levels of NOx compared to those with Cs (Figure 7b), due to the fact that curcumin encapsulated in nanocarriers has increased the antioxidant effect, as previously demonstrated [38,39]. The serum level of TOS was reported to be increased in patients with chronic ischemic heart failure [40]. For patients with acute MI it is an essential factor correlated with the complexity and intensity of coronary artery disease [41]. Curcumin was reported to have a modulatory role on oxidative status [42], which can explain the decreased levels of TOS in the myocardial tissue. 

According to our results, thiol and TAC were reduced in the myocardial tissue after ISO administration, but increased levels were found in rats pre-treated with Cs and nC (Figure 8a,b). Thiols function is to serve as the reductant of toxic peroxides since intracellular thiols and unsaturated lipid are the major targets of ROS [43,44]. Thiols represent not only the most vulnerable targets of oxygen reactive species and related oxidants, but they are also a robust and a versatile defence system against biochemical alterations induced by oxidative stress [44]. In physiological conditions, low levels of oxidants selectively oxidase some protein thiols and such oxidative changes play an important role in cellular metabolism, signal transduction, proliferation and cell death [44]. Inhibition of the NF-kappaB activation and induction of glutathione biosynthesis could be the underlying mechanism for the higher tissular level of thiol after curcumin administration [45,46]. TAC of myocardial tissue is used for the measurement of the heart ability to defend against ROS [31]. TAC was reported to have low levels in patients with MI, therefore drugs with an antioxidant effect may have beneficial effects in preventing coronary artery diseases [47]. Grater results obtained after curcumin nanoparticle pre-treatment, not only for thiols but also for TAC, are explained by increased metabolic stability and the better tissue distribution of curcumin nanoparticles [48,49].

Rats with induced MI had higher levels of LDH (Figure 4) AST and ALT (Figure 5a,b). The prior administration of Cs and nC was found to considerably reduce the ISO-induced raise in the serum activities of LDH (Figure 4), AST and ALT (Figure 5a,b). LDH has an increased serum activity following MI within 6–12 h after the onset of myocardial ischemia, has a peak at about 48 h and it remains elevated for 4–14 days before returning to normal values. Its use in the diagnosis of MI is limited due to its non-specificity, and increased levels are found in different muscle disorders or anaemia [50]. AST is an enzyme found mainly in the liver, heart, and muscles and is released into the blood in case of damage of these organs [51]. The levels of AST activity begin to rise in the serum at 3–8 h after the onset of the myocardial injury with a peak level at 24 h and complete clearance in 3 to 6 days [51]. Previously AST was considered as a very good marker of cardiac injury, but due to low its low specificity, it has a limited utility in diagnosis of MI [51]. ALT is an enzyme primarily concentrated in hepatocytes and renal tubular epithelium, with activity present in cardiac and skeletal muscle. In MI its levels rise in 6–10 h and remain high for about 4 days [51]. Even if ALT has low activity in the human heart, despite the modest increases in ALT in plasma after acute MI, it is useful to estimate the size of infarcted tissue [21]. It was previously reported that due to its antioxidant and anti-inflammatory properties, curcumin preserves the normal histological architecture of the heart and minimizes elevation of plasma cardiac enzyme markers, such as creatine kinase and creatine kinase MB, following experimental MI by ISO administration [52]. It was also proved that curcumin reduces the release of LDH and increases cell viability in case of cardiomyocytes injury [53]. Curcumin and curcumin nanoparticles pre-treatment were proved to have a hepato-protective effect in high fructose diet or streptozotocin-induced diabetes mellitus, by preventing ALT and AST elevation [54]. Swamy et al. also reported that curcumin has a cellular membrane stabilizing property [55], since curcumin pre-treatment inhibited myocardial damage by preventing elevation of AST and ALT after ISO administration. The better cardioprotective effect offered by nC is due to improved bioavailability of curcumin nanoparticles, which improves the delivery of the drug to the infarcted myocardial tissue [34]. 

In our study, rats with ISO-induced MI had a higher level of glucose (Figure 6). Acute hyperglycaemia is a common feature during the early phase of acute MI, even in patients without a history of diabetes mellitus [56]. Even more, it is associated with markedly increased mortality since acute hyperglycaemia exaggerates inflammation by the oxidative mechanism in MI [56]. An increased plasma glucose level has been reported to be capable of inducing electrophysiological alterations in myocardial tissue that might trigger fatal arrhythmias [57]. The underlying mechanism for this is that acute increase of glycaemia in normal subjects produces a significant elongation of the QT interval, increasing the risks for arrhythmias with fatal outcome [57]. Curcumin reduces glycaemia level by stimulating insulin secretion [58]. Increased distribution of the curcumin nanoparticles in the body organs can explain the better results obtained for nC [59].

As previously reported by our group, in the group with ISO-induced MI, histopathological examination showed marked ultrastructural alterations with diffuse infiltration with leukocytes, severe interstitial oedema, and extended sub-endocardial necrosis [20]. Rats pre-treated with Cs and nC had less severe myocardial tissue changes with less severe interstitial oedema, reduced accumulation of inflammatory cells and less extended myofibrillary degeneration and necrosis, with the best results for nC in the dose of 200 mg/kg bw [20]. Curcumin pre-treatment prevented the myocardial injury by reducing the systemic inflammatory responses, with decreasing serum levels of the several inflammatory cytokines: IL (interleukin) -1α, IL-1β, IL-6, TNF-α (tumour necrosis factor alpha), and RANTES (regulated upon activation, normal T cell expressed and secreted) [20]. Oedema is prevented due to curcumin’s ability to curtail the eicosanoids-induced vasodilation and to reduce vascular permeability at the site of myocardial injury [60]. Curcumin has also an important role in improving the mitochondrial function of the injured cardiomyocytes which can explain the reduction of myocardial cell degradation and apoptosis [61]. Higher bioavailability attributed to the increased absorption of curcumin nanoparticles through the gastrointestinal tract, decreased degradation, and reduced clearance in the myocardial tissue can explain the increased tissular protective effects observed on nC compared to Cs [62].

The present study demonstrates that curcumin and curcumin nanoparticles pre-treatment can prevent extension of damaged myocardial tissue after MI, with better effect expressed by the smallest difference as compared with the control group (no myocardial infarction, Figure 9 and Figure 10). When the curcumin nanoparticle solution was compared to the standard curcumin solution, the best effects were observed for the highest concentration (nC200, Figure 9 and Figure 10). The mechanism by which curcumin nanoparticles exert better cardioprotective effects involves their increased ability to reduce oxidative stress in the myocardial tissue and to enhance antioxidant defence. Curcumin nanoparticles can be taken into consideration as part of the preventive strategies in cardiovascular diseases since the early onset of this therapy can be an essential option for preventing the extension of myocardial tissue damage in case acute myocardial infarction.

### Study Limitations and Call for Future Researches

No measurements of curcumin and curcumin nanoparticles concentrations in the blood or heart tissue were done in this study since such quantifications were out of our aim. Since the beneficial effects of curcumin and curcumin nanoparticles were demonstrated, future studies could be conducted to measure the concentration of curcumin and curcumin nanoparticles in both blood and heart tissue. Even more, to evaluate in more extent their expressions as well as the relation between the effects and blood and/or tissue concentrations is of real interest. Such measurement could bring more insights regarding curcumin and curcumin nanoparticles bioavailability and metabolism in the myocardium. Furthermore, the presence and/or quantification of curcumin and curcumin nanoparticles on the histological slices could also be conducted. 

## 4. Materials and Methods 

### 4.1. Ethics Statement

All work was conducted in accordance with the Declaration of Helsinki on Animal Studies. The experimental protocol was approved by the Ethics Committee of the “Iuliu Hațieganu” University of Medicine and Pharmacy Cluj-Napoca (certificate number 53/22.01.2018) and also by the Sanitary-Veterinary and Food Safety Directorate from Cluj-Napoca (certificate number 99/21.02.2018). The experimental protocol was in adherence to international and national guidelines for the care and use of animals.

### 4.2. Chemicals and Reagents 

Isoproterenol hydrochloride (ISO) (98%) together with conventional curcumin powder (Cs) (≥94% curcuminoid content; ≥80% Curcumin) were purchased from Sigma–Aldrich (St. Louis, MO, USA). Curcumin nanoparticles (nC) were purchased from CVI Pharma (Vietnam). In the nC, curcumin, the active compound, is enclosed in polymer-based nanoparticles with size between 30 nm and 100 nm. All other chemicals used were of analytical grade.

### 4.3. Animal Grouping 

A total of fifty-six white female Wistar-Bratislava rats (200–250 g) from the Animal Department of Faculty of Medicine, Iuliu Haţieganu University of Medicine and Pharmacy Cluj-Napoca, were used in the study. During the experiment the animals were kept in polypropylene cages and acclimated at standard environmental conditions of 22–24°C, humidity 55 ± 15% and 12 h/12 h light/dark cycle at the Department of Pathophysiology. Animals had free access to food (standard pellets) as basal diet and water ad libitum.

### 4.4. Myocardial Ischemia Induction

The rats were randomly divided into eight groups of seven animals each and treated as follows: 

(1) Group 1 (C), the control group, rats were pre-treated with saline;

(2) Group 2 (MI-C), the myocardial infarction model group, rats were pre-treated with saline and MI was induced; 

(3) Group 3 (Cs100), rats were pre-treated with conventional curcumin in a dose of 100 mg/kg bw and MI was induced;

(4) Group 4 (Cs150), rats were pre-treated with conventional curcumin in a dose of 150 mg/kg bw and MI was induced;

(5) Group 5 (Cs200), rats were pre-treated with conventional curcumin in a dose of 200 mg/kg bw and MI was induced;

(6) Group 6 (nC100), rats were pre-treated with curcumin nanoparticles in a dose of 100 mg/kg bw and MI was induced;

(7) Group 7 (nC150), rats were pre-treated with curcumin nanoparticles in a dose of 150 mg/kg bw and MI was induced;

(8) Group 8 (nC200), rats were pre-treated with curcumin nanoparticles in a dose of 200 mg/kg bw and MI was induced

**Figure 11:**
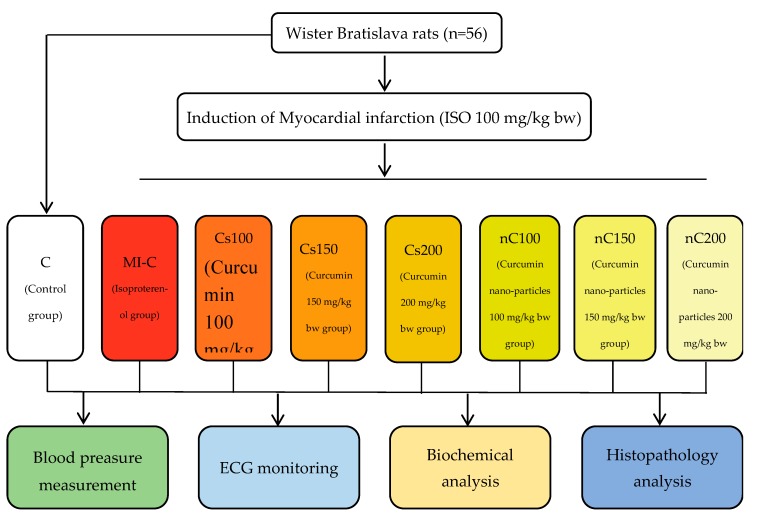
Flow chart demonstrating the experimental study groups.

A flowchart of the experimental study groups involved in this study is shown in Figure 11.

To induce myocardial infarction, rats from groups 2 to 8 were subcutaneously injected with a dose of 100 mg/kg body weight for two days at 24 h interval (on day 13th and 14th), following the model described by Soraya et al. [63]. It was previously demonstrated that the dose of 100 mg/kg of ISO can be used to induce infarct-like lesion on ECG, biological and histological changes characteristic for MI [64]. MI was successfully induced, with elevation of creatine kinase and creatine kinase MB, as it was previously reported [20]. The first group (control group) received subcutaneously injected saline following the schedule of the groups with MI.

Curcumin and curcumin nanoparticles were dissolved in peanut oil. Cs and nC solutions were administrated orally, by gavage. Pre-treatment with curcumin for 15 days was previously demonstrated to provide cardiovascular protection in case of acute myocardial infarction [20,65].

### 4.5. Blood Pressure Measurement 

**Figure 12:**
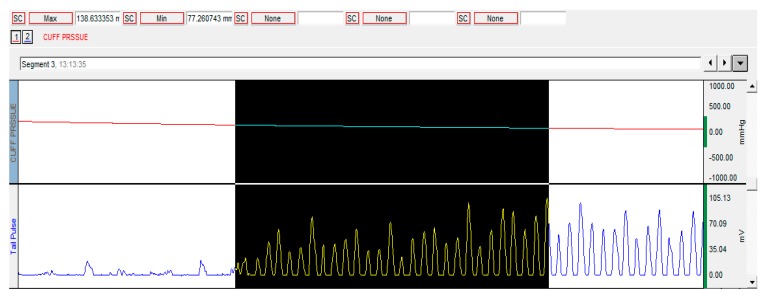
Blood pressure measurement method.

The systolic and diastolic arterial blood pressures in conscious, non-anaesthetized rats were measured using a tail-cuff plethysmography of a Biopac MP36 system with NIBP200A extension (Goleta, CA, USA) as shown in Figure 12, following the method described by Bulboaca et al. [66]. The blood pressures were monitored on day 0 (at the beginning of the experiment), on day 12 (at 24 h before ISO administration) and on day 15 (at 24 h after the last dose of ISO). 

### 4.6. Electrocardiography Monitoring

Electrocardiography (ECG) was performed using the method described by Balea at al. [23]. Animals were under general anaesthesia with xylazine (2.6 mg/kg, i.p.) and ketamine (26 mg/kg, i.p.). 15 min after anaesthesia induction, animals were placed on a board, in the supine position. Electrodes were attached to the paw pads of each rat, and ECG was record in lead II using Biopac MP36 system (Goleta, CA, USA). ECG analysis was performed using Biopac Student Lab 3.7.7 (Goleta, CA, USA) and included calculation of the RR intervals (msec), PR segments (msec), QRS duration (msec), QT intervals (msec), corrected QT intervals (QTc) (msec), according to Bazett formula [67], and ST segment changes (mV). The heart rate (HR) (beats/min) was calculated from the RR interval according to the following formula: HR = 60,000/RR [67].

### 4.7. Blood Samples and Serum Analysis 

On day 15, at 24 h after the administration of the last dose of ISO, under light ketamine anaesthesia with xylazine and ketamine, the blood samples were collected from the retro-orbital plexuses of each rat. At the end of the experiment, the rats were sacrificed by an overdose of anaesthetics. Serum levels of the levels of lactate dehydrogenase (LDH), aspartate transaminase (AST) and alanine transaminase (ALT) were measured using Jasco V-530 UV-Vis spectrophotometer (Jasco International Co. Ltd., Tokyo, Japan). 

### 4.8. Preparation of Tissue Homogenate and Myocardial Oxidative Stress Evaluation

Prior analysis, tissue was weighted and homogenized in four volumes of phosphate-buffered saline solution. The homogenization was made at 27,000 rpm using and automated Witeg Homogenizer (HG-15D, Wertheim, Germany). The homogenate was centrifuged (15,000 rpm for 15 min at 4 °C) and the clear supernatant was used for oxidative stress analysis. Oxidative stress in the myocardial tissue was assessed by measuring five parameters: malondialdehyde (MDA), the indirect assessment of NO synthesis (NOx), and total oxidative status (TOS) for the evaluation of oxidative stress intensity. Thiol and total antioxidative capacity (TAC) were measured to evaluate the antioxidant capacity. All the oxidative stress parameters measurements were performed using a Jasco V-350 UV-VIS spectrophotometer (Jasco International Co., Ltd., Tokyo, Japan).

### 4.9. Statistical Analysis

Measured data were expressed as mean and standard deviation. The groups’ differences in blood pressure, ECG, and biological changes were assessed with the Mann–Whitney test. The differences in the values relative to the control group (the C group, no MI) of the serum measurements and oxidative stress parameters were investigated to evaluate the efficacy of the nC. The differences were represented using box and whiskers plots with the median as the middle point, the values of 25th and 75th percentile as the box, and the minimum and maximum value as the whiskers. The circle in the box and whiskers plot indicate an outlier and the star indicate an extreme value. The Kruskal Wallis test was applied on the differences and the *p*-values smaller than 0.007 were considered statistical significant. Statistical analysis was performed with Statistica software (v. 8, StatSoft, Tusla, OK, USA).

## 5. Conclusionss

Taken together, the findings of our study demonstrate that curcumin nanoparticles exert grater antioxidative effects than conventional curcumin by suppressing myocardial tissue oxidative stress and enhancing anti-oxidative activity. Other cardio-protective effects offered by curcumin, with better results for curcumin nanoparticles, are evidenced by prevention of ECG alterations in case of isoproterenol-induced myocardial infarction. This study provides a novel strategy into curcumin nanoparticles-induced enhanced cardioprotection, which may be helpful in limiting myocardial damage extension in the case of myocardial infarction.

## Figures and Tables

**Table 1:** Dynamic evolution of systolic and diastolic blood pressure from baseline to 15 days follow-up.

Group abb.	Day 0 (baseline)		Day 12 (before ISO Administration)		Day 15 (after ISO Administration)	
	SBP (mmHg)	DBP (mmHg)	SBP (mmHg)	DBP (mmHg)	SBP (mmHg)	DBP (mmHg)
C	136.29 (4.92)	68.43 (2.88)	136.29 (2.81)	68.71 (1.80)	136.00 (3.74)	69.57 (2.88)
MI-C	137.14 (2.41)	68.86 (2.12)	135.00 (4.73)	68.29 (2.14)	137.00 (4.65)	70.43 (2.37)
Cs100+ISO	136.14 (5.58)	69.57 (2.37)	138.85 (2.91)	69.42 (2.81)	138.71 (4.03)	67.86 (4.38)
Cs150+ISO	134.57 (3.60)	66.14 (2.34)	134.57 (5.25)	66.42 (3.69)	134.43 (4.28)	69.00 (1.41)
Cs200+ISO	134.71 (3.54)	68.29 (1.50)	135.71 (6.67)	66.29 (2.98)	133.00 (2.08)	69.57 (1.81)
nC100+ISO	136.00 (3.60)	68.57 (1.90)	134.85 (3.33)	68.00 (2.58)	136.00 (4.97)	68.57 (2.44)
nC150+ISO	136.28 (3.64)	67.14 (3.80)	134.43 (5.47)	67.57 (2.43)	134.00 (8.29)	70.71 (3.64)
nC200+ISO	136.14 (2.85)	67.71 (2.87)	136.14 (4.29)	68.85 (1.57)	137.14 (2.97)	69.71 (2.06)

The values in the body of the table represent the mean and standard deviation (in the round brackets); SBP = systolic blood pressure; DBP = diastolic blood pressure = diastolic blood pressure; C = Control; MI-C = MI without any pre-treatment; Cs = curcumin solution, as 100 mg/kg bw (Cs100), 150 mg/kg bw (Cs150), 200 mg/kg bw (Cs200); nC = curcumin nano-particle solution as 100 mg/kg bw (nC100), 150 mg/kg bw (nC150), and 200 mg/kg bw (nC200); ISO = isoproterenol.

**Table 2:** Initial electrocardiogram (ECG) characteristics by group.

Group abb.	RR(ms)	HR(b/min)	PR(ms)	QRS(ms)	QT(ms)	QTc(ms)	ST(mV)
C	210 (5.74)	285 (7.73)	43 (2.99)	35 (3.55)	75 (3.78)	64 (3.40)	0.11 (0.04)
MI-C	204 (11.56)	294 (16.49)	44 (2.99)	36 (3.25)	75 (4.82)	65 (4.97)	0.10 (0.01)
Cs100+ISO	211 (9.27)	285 (12.37)	44 (3.68)	36 (3.41)	76 (4.86)	64 (3.15)	0.10 (0.03)
Cs150+ISO	211 (8.38)	285 (10.96)	44 (2.91)	37 (4.76)	76 (5.16)	64 (3.70)	0.10 (0.03)
Cs200+ISO	206 (9.04)	292 (13.09)	42 (2.23)	37 (3.15)	77 (4.11)	66 (4.30)	0.11 (0,03)
nC100+ISO	209 (8.51)	288 (11.53)	41 (1.63)	34 (1.15)	75 (2.91)	63 (3.43)	0.12 (0.03)
nC150+ISO	210 (4.69)	286 (6.43)	41 (1.51)	36 (3.26)	73 (2.81)	62 (2.61)	0.11 (0.03)
nC200+ISO	209 (7.63)	288 (10.91)	43 (1.95)	36 (2.43)	77 (1.72)	66 (2.19)	0.10 (0.01)

The values in the body of the table are means and standard deviation (in the round brackets); RR = RR interval; HR = heart rate; PR = PR interval; QRS = QRS complex, QT = QT interval; QTc = corrected QT interval; ST = ST segment; C = Control; MI-C = MI without any pre-treatment; Cs = curcumin solution, as 100 mg/kg bw (Cs100), 150 mg/kg bw (Cs150), 200 mg/kg bw (Cs200); nC = curcumin nano-particle solution as 100 mg/kg bw (nC100), 150 mg/kg bw (nC150), and 200 mg/kg bw (nC200); ISO = isoproterenol.

**Table 3:** ECG characteristics by group before isoproterenol (ISO) administration.

Group abb.	RR (ms)	HR (b/min)	PR (ms)	QRS (ms)	QT (ms)	QTc (ms)	ST (mV)
C	207 (6.35)	291 (8.93)	43 (2.93)	36 (2.93)	76 (1.63)	65 (1.71)	0.11 (0.02)
MI-C	210 (6,34)	286 (8.55)	44 (1.46)	36 (3.04)	77 (3.39)	65 (3.73)	0.10 (0.01)
Cs100+ISO	213 (5.40)	282 (7.21)	43 (1.51)	35 (2.29)	78 (2.37)	65 (2.58)	0.09 (0.03)
Cs150+ISO	218 (3.72)	275 (4.69)	43 (1.57)	37 (2.41)	75 (2,69)	62 (2.30)	0.10 (0.02)
Cs200+ISO	225 (7.62)	267 (8.92)	43 (2.12)	36 (3.15)	76 (3,35)	61 (3.25)	0.10 (0.02)
nC100+ISO	225 (2.37)	266 (2.79)	42 (1.27)	35 (1.89)	75 (4,06)	61 (3.32)	0.11 (0.02)
nC150+ISO	232 (1.99)	258 (2.21)	43 (2.04)	35 (1.72)	75 (2,73)	60 (2.11)	0.10 (0.02)
nC200+ISO	244 (3.27)	246 (3.29)	44 (2.76)	35 (2.23)	78 (1.99)	61 (1.76)	0.10 (0.03)

The values represent the mean and standard deviation (in the round brackets); RR = RR interval; HR = heart rate; PR = PR interval; QRS = QRS complex, QT = QT interval; QTc = corrected QT interval; ST = ST segment; C = Control; MI-C = MI without any pre-treatment; Cs = curcumin solution, as 100 mg/kg bw (Cs100), 150 mg/kg bw (Cs150), 200 mg/kg bw (Cs200); nC = curcumin nano-particle solution as 100 mg/kg bw (nC100), 150 mg/kg bw (nC150), and 200 mg/kg bw (nC200); ISO = isoproterenol.

**Table 4:** ECG characteristics by group after ISO administration.

Group abb.	RR (ms)	HR (b/min)	PR (ms)	QRS (ms)	QT (ms)	QTc (ms)	ST (mm)
C	205 (3.60)	292 (5.11)	42 (1.91)	35 (3.67)	75 (1.51)	64 (1.28)	0.11 (0.04)
MI-C	177 (4.75)	339 (9.12)	45 (2.79)	50 (2.37)	114 (7.46)	105 (7.3)	−0.06 (0.03)
Cs100+ISO	207 (3.35)	290 (4.68)	43 (1.50)	49 (1.11)	107 (2.87)	91 (2.97)	−0.04 (0.01)
Cs150+ISO	212 (3.10)	284 (4.14)	42 (3.34)	41 (2.07)	103 (2.94)	87 (2.61)	−0.03 (0.02)
Cs200+ISO	219 (2.56)	274 (3.18)	44 (2.34)	39 (1.27)	98 (2.61)	81 (2.5)	−0.01 (0.01)
nC100+ISO	227 (3.15)	265 (3.75)	42 (2.43)	37 (2.04)	96 (3.34)	78 (2.37)	0 (0.02)
nC150+ISO	235 (3.21)	256 (3.51)	44 (2.98)	37 (1.21)	87 (3.86)	69 (3,06)	0.01 (0.01)
nC200+ISO	248 (3.55)	242 (3.48)	43 (2.06)	35 (1.35)	82 (3.95)	64 (2.91)	0.04 (0.02)

The values represent the mean and standard deviation (in the round brackets); RR = RR interval; HR = heart rate; PR = PR interval; QRS = QRS complex, QT = QT interval; QTc = corrected QT interval; ST = ST segment; C = Control; MI-C = MI without any pre-treatment; Cs = curcumin solution, as 100 mg/kg bw (Cs100), 150 mg/kg bw (Cs150), 200 mg/kg bw (Cs200); nC = curcumin nano-particle solution as 100 mg/kg bw (nC100), 150 mg/kg bw (nC150), and 200 mg/kg bw (nC200); ISO = isoproterenol.

## Supplementary Materials

The supplementary materials are available online.
