# Supplementary material for: Curcumin Nanoparticles Protect against Isoproterenol Induced Myocardial Infarction by Alleviating Myocardial Tissue Oxidative Stress, Electrocardiogram, and Biological Changes
Source: Molecules. 2019 Aug 1;24(15):2802. doi: 10.3390/molecules24152802 (PMC6696485; doi:10.3390/molecules24152802)
Supplement: Supplementary file 1 [file molecules-24-02802-s001.pdf]

Curcumin nanoparticles protect against isoproterenol induced myocardial infarction by alleviating myocardial tissue oxidative stress, electrocardiogram, and biological changes

Paul-Mihai Boarescu, Ioana Boarescu, Ioana Corina Bocșan, Raluca Maria Pop, Dan Gheban, Adriana Elena Bulboacă, Cristina Nicula, Ruxandra-Mioara Râjnoveanu and Sorana D. Bolboacă

Table S1. P-values for comparisons between the study groups for electrocardiogram monitoring.

	**MI-C vs. C**	**Cs100+ ISO vs. MI-C**	**Cs150+ ISO vs. MI-C**	**Cs200+ ISO vs. MI-C**	**nC100+ ISO vs. MI-C**	nC150+ ISO vs. MI-C	nC200+ ISO vs. MI-C	Cs xxx+ISO vs. Cs yyy+ISO			nC xxx+ISO vs. nC yyy+ISO			nC xxx+ISO vs. Cs xxx+ISO		
								150 vs. 100	200 vs. 100	200 vs. 150	150 vs. 100	200 vs. 100	200 vs. 150	100 vs. 100	150 vs. 150	200 vs. 200
RR(ms) day 0	0.3067	0.3067	0.3379	0.8480	0.6093	0.1797	0.3711	0.7983	0.4822	0.4062	0.6547	0.7015	0.9491	0.7015	0.7494	0.4433
HR(b/min) day 0	0.3067	0.3067	0.3379	0.8480	0.6093	0.1797	0.3711	0.7983	0.4822	0.4062	0.6547	0.7015	0.9491	0.7015	0.7494	0.4433
PR(ms) day 0	0.5653	0.7983	0.7983	0.1797	0.1102	0.2013	0.5229	0.7015	0.2013	0.0845	0.4433	0.1102	0.2013	0.1417	0.0845	0.2013
QRS (ms) day 0	0.4062	1.0000	0.6547	0.5229	0.1417	0.8983	0.5653	0.7015	0.6093	1.0000	0.3067	0.0253	0.9491	0.1102	0.7983	0.8480
QT(ms) day 0	0.8983	0.8983	0.6547	0.4822	0.6547	0.3067	0.5653	1.0000	0.6093	1.0000	0.4822	0.1102	0.0152	0.7494	0.4433	0.8983
QTc(ms) day 0	0.5653	0.7494	0.6547	0.7494	0.5653	0.2774	0.7494	0.7494	0.4062	0.4822	0.6547	0.1417	0.0350	0.4062	0.3379	0.7983
ST(mV) day 0	0.5229	0.5653	0.7983	0.7983	0.5653	0.6547	0.2502	0.9491	0.5653	0.5229	0.7494	0.1417	0.2013	0.2774	0.6093	0.4433
RR(ms) day 12	0.4062	0.2502	0.0253	0.0049	0.0017	0.0017	0.0017	0.0736	0.0073	0.0736	0.0017	0.0017	0.0017	0.0017	0.0017	0.0017
HR(b/min) day 12	0.4062	0.2502	0.0253	0.0049	0.0017	0.0017	0.0017	0.0736	0.0073	0.0736	0.0017	0.0017	0.0017	0.0017	0.0017	0.0017

PR(ms) day 12	0.3067	0.6093	0.2248	0.2013	0.0553	0.3711	0.3067	0.4822	0.5653	0.9491	0.4062	0.4822	0.9491	0.2013	0.7983	0.7983
QRS (ms) day 12	0.6547	0.8983	0.3711	0.8983	0.9491	1.0000	0.9491	0.2248	0.7015	0.4822	0.8983	0.8480	0.8983	1.0000	0.2248	0.9491
QT(ms) day 12	0.2248	0.8480	0.2248	0.4433	0.3067	0.1797	0.8983	0.1599	0.1797	0.9491	1.0000	0.2013	0.0967	0.2502	1.0000	0.2013
QTc(ms) day 12	0.4062	0.4822	0.1417	0.1102	0.0350	0.0350	0.0639	0.0639	0.0967	0.4822	0.4822	0.6547	0.5653	0.0350	0.0845	0.7494
ST(mV) day 12	0.8480	0.3711	0.7494	0.7015	0.6547	0.4062	0.9491	0.5229	0.7015	0.8983	0.2013	0.7015	0.5229	0.2248	0.7015	0.7494
RR(ms) day 15	0.0017	0.0017	0.0017	0.0017	0.0017	0.0017	0.0017	0.0476	0.0017	0.0022	0.0017	0.0017	0.0017	0.0017	0.0017	0.0017
HR(b/min) day 15	0.0017	0.0017	0.0017	0.0017	0.0017	0.0017	0.0017	0.0476	0.0017	0.0022	0.0017	0.0017	0.0017	0.0017	0.0017	0.0017
PR(ms) day 15	0.0476	0.1252	0.0967	0.3711	0.1599	0.7015	0.1599	0.3711	0.4062	0.2248	0.1797	0.6547	0.3067	0.7494	0.2013	0.2774
QRS (ms) day 15	0.0017	0.1252	0.0017	0.0017	0.0017	0.0017	0.0017	0.0017	0.0017	0.0639	0.7983	0.0639	0.0350	0.0017	0.0022	0.0017
QT(ms) day 15	0.0017	0.0253	0.0027	0.0017	0.0017	0.0017	0.0017	0.0476	0.0017	0.0088	0.0027	0.0017	0.0476	0.0017	0.0017	0.0017
QTc(ms) day 15	0.0017	0.0017	0.0017	0.0017	0.0017	0.0017	0.0017	0.0350	0.0017	0.0027	0.0017	0.0017	0.0127	0.0017	0.0017	0.0017
ST(mV) day 15	0.0017	0.2502	0.0639	0.0106	0.0073	0.0017	0.0017	0.2774	0.0215	0.5653	0.4433	0.0049	0.0073	0.0152	0.0088	0.0017

Table S2. P-values for comparisons between the study groups for of lactate dehydrogenase (LDH), aspartate transaminase (ASAT), alanine transaminase (ALAT), and glycemia.

	**MI-C vs. C**	**Cs100+ ISO vs. MI-C**	**Cs150+ ISO vs. MI-C**	**Cs200+ ISO vs. MI-C**	**nC100+ ISO vs. MI-C**	**nC150+ ISO vs. MI-C**	nC200+ ISO vs. MI-C	Cs xxx+ISO vs. Cs yyy+ISO			nC xxx+ISO vs. nC yyy+ISO			nC xxx+ISO vs. Cs xxx+ISO		
								150 vs. 100	200 vs. 100	200 vs. 150	150 vs. 100	200 vs. 100	200 vs. 150	100 vs. 100	150 vs. 150	200 vs. 200
		α1	α2	α3	α4	α5	α6	β1	β2	γ1	λ1	λ2	μ1	β3	γ2	ε1
LDH (U/L)	0.0017	0.0017	0.0017	0.0017	0.0017	0.0017	0.0017	0.0040	0.0017	0.0845	0.0017	0.0017	0.0181	0.0017	0.0017	0.0017
ASAT (U/L)	0.0017	0.0017	0.0017	0.0017	0.0017	0.0017	0.0017	0.0017	0.0017	0.0060	0.0027	0.0017	0.0476	0.0017	0.0017	0.0017
ALAT (U/L)	0.0017	0.0017	0.0017	0.0017	0.0017	0.0017	0.0017	0.0181	0.0127	0.4822	0.0017	0.0017	0.0017	0.0073	0.0017	0.0017
Glycemia (mg/dL)	0.0017	0.0017	0.0017	0.0017	0.0017	0.0017	0.0017	0.0106	0.0049	0.3711	0.0476	0.0017	0.0553	0.0027	0.0088	0.0017

Table S3. P-values for comparisons between the study groups for oxidative stress parameters in myocardial tissue.

	**MI-C vs. C**	**Cs100+ ISO vs. MI-C**	**Cs150+ ISO vs. MI-C**	**Cs200+ ISO vs. MI-C**	**nC100+ ISO vs. MI-C**	**nC150+ ISO vs. MI-C**	nC200+ ISO vs. MI-C	Cs xxx+ISO vs. Cs yyy+ISO			nC xxx+ISO vs. nC yyy+ISO			nC xxx+ISO vs. Cs xxx+ISO		
								150 vs. 100	200 vs. 100	200 vs. 150	150 vs. 100	200 vs. 100	200 vs. 150	100 vs. 100	150 vs. 150	200 vs. 200
		α1	α2	α3	α4	α5	α6	β1	β2	γ1	λ1	λ2	μ1	β3	γ2	ε1
MDA [nmol/L]	0.0017	0.0060	0.0026	0.0017	0.0017	0.0017	0.0017	0.0350	0.0017	0.0181	0.0088	0.0017	0.5224	0.0017	0.0017	0.0017
NOx [μmol/L]	0.0017	0.0027	0.0017	0.0017	0.0017	0.0017	0.0017	0.1797	0.0639	0.4428	0.3352	0.0039	0.0040	0.0030	0.0030	0.0017
TOS [μmol H2O2 equiv./L]	0.0017	0.0027	0.0017	0.0017	0.0017	0.0017	0.0017	0.0474	0.0253	0.7009	0.2003	0.0060	0.0405	0.0027	0.0027	0.0033
Thiol [mmol/L]	0.0017	0.0250	0.0040	0.0017	0.0017	0.0016	0.0017	0.1768	0.0070	0.0403	0.0450	0.0033	0.0080	0.0017	0.0017	0.0017
TAC [mmol Trolox/L]	0.0016	0.0016	0.0016	0.0016	0.0015	0.0016	0.0016	0.0250	0.0017	0.0026	0.0038	0.0016	0.0017	0.0016	0.0016	0.0017
